# Recombinant expression and purification of the 2,5-diketocamphane 1,2-monooxygenase from the camphor metabolizing *Pseudomonas putida *strain NCIMB 10007

**DOI:** 10.1186/2191-0855-1-13

**Published:** 2011-06-23

**Authors:** Maria Kadow, Stefan Saß, Marlen Schmidt, Uwe T Bornscheuer

**Affiliations:** 1 Department of Biotechnology and Enzyme Catalysis, Institute of Biochemistry, Greifswald University, Felix-Hausdorff-Str. 4, D-17487 Greifswald, Germany

**Keywords:** Baeyer-Villiger monooxygenases, camphor, *Pseudomonas putida* NCIMB 10007, 2,5-diketocamphane 1,2-monooxygenase, bicyclic ketones

## Abstract

Three different Baeyer-Villiger monooxygenases (BVMOs) were reported to be involved in the camphor metabolism by *Pseudomonas putida *NCIMB 10007. During (+)-camphor degradation, 2,5-diketocamphane is formed serving as substrate for the 2,5-diketocamphane 1,2-monooxygenase. This enzyme is encoded on the CAM plasmid and depends on the cofactors FMN and NADH and hence belongs to the group of type II BVMOs. We have cloned and recombinantly expressed the oxygenating subunit of the 2,5-diketocamphane 1,2-monooxygenase (2,5-DKCMO) in *E. coli *followed by His-tag-based affinity purification. A range of compounds representing different BVMO substrate classes were then investigated, but only bicyclic ketones were converted by 2,5-DKCMO used as crude cell extract or after purification. Interestingly, also (-)-camphor was oxidized, but conversion was about 3-fold lower compared to (+)-camphor. Moreover, activity of purified 2,5-DKCMO was observed in the absence of an NADH-dehydrogenase subunit.

## Introduction

**Figure 1:**
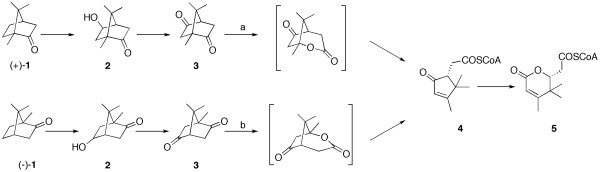
**Camphor degradation in *Pseudomonas putida *NCIMB 10007:** In the first step camphor (1) is hydroxylated by the P450_Cam_-monooxygenase (Unger et al. 1986) followed by an oxidation by the 5-exo-alkohol dehydrogenase (Koga et al. 1989) yielding the corresponding diketocamphane (3). (+)-**1** is degraded by the 2,5-dicetocamphane 1,2-monooxygenase (a), while (-)-**1** requires the 3,6-diketocamphane 1,6-monooxygenase (b). Both resulting lactones are unstable and lead to spontaneous formation of the 2-oxo-Δ3-4,5,5-trimethylcylopentenylacetic acid, which is further converted to a coenzyme A derivative (4), which is again a substrate for a third involved BVMO (2-oxo-Δ3-4,5,5-trimethylcylopentenylacetic acid monooxygenase, often designated as MO2) (Ougham et al. 1983).

**Figure 2:**
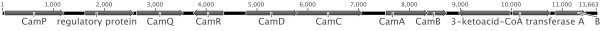
**Operon of the CAM-plasmid: **CamA: putidaredoxin reductase (M12546.1); CamB: putidaredoxin (J05406.1); CamC: cytochrome P-450cam (M12546.1); CamD: 5-exo-alkohol-dehydrogenase (M13471.1); CamP: 1,2-diketocamphane 2,5-monooxygenase (AY450285.1); CamQ: lactone hydrolase (AY450285); CamR: regulatory protein. The putative 3-ketoacid-CoA-transferases A and B were identified in this work by gene-walking PCR.

The discovery of the enzymatic Baeyer-Villiger reaction is closely connected to the exploration of the biodegradation of camphor (**1**) in Pseudomonads (Figure 1). Initial studies on the microbial decomposition of (+)-**1 **by *Pseudomonas putida *NCIMB 10007 isolated from sewage sludge were already carried out in 1959 (Bradshaw et al. 1959) and the involved enzymes were separated and characterized during the following decade. In studies of the enzymatic lactonization of the intermediate 2,5-diketocamphane (**3**) from the (+)-camphor-grown organism it was shown that two enzyme fractions were responsible for the Baeyer-Villiger-monooxygenase (BVMO) catalyzed reaction step (Conrad et al. 1961). The first enzyme turned out to be a FMN-coupled NADH-dehydrogenase [EC 1.6.8.1], while the second subunit was claimed to be a ketolactonase. Since mechanistic similarities to the chemical Baeyer-Villiger oxidation of bicyclic ketones (Meinwald and Frauenglass 1960) were detected, the nomenclature of the ketolactonase was changed to a BVMO. In 1965 a second lactonizing system for the degradation of (-)-**1 **was found (Conrad et al. 1965a). Thus it was claimed that (+)-**1 **and its derivatives were only converted by the (+)-camphor induced 2,5-diketocamphane 1,2-monooxygenase (2,5-DKCMO), while (-)-**1 **is converted by the (-)-camphor induced 3,6-diketocamphane 1,6-monooxygenase (Jones et al. 1993) (Figure 1). Later it was claimed, that whichever enantiomer of camphor is given to the growth medium, both diketocamphane monooxygenases are induced (Gagnon et al. 1994). The ability to decompose camphor turned out to be inducible in several fluorescent Pseudomonads, where most of the involved enzymes, including both type II monooxygenases, are located on a 230 kb (165 MDa) plasmid (CAM plasmid, Figure 2) (Chakrabarty 1976).

During the 1990s, several studies on the conversion of cyclic and bicyclic alkanones using whole *Pseudomonas putida *cells or partially purified enzymes of this organism were performed. Regarding the evolutionary predisposition of the three BVMOs involved in camphor metabolism, diketocamphane monooxygenases turned out to catalyze the efficient production of optically active bicyclic lactones in an enantiodivergent and highly selective manner (Gagnon et al. 1994). Especially bicyclo [3.2.0.] ketones and norcamphor-derived compounds were investigated and benzyloxylactone, achieved from a norcamphor derivative, emerged as an important precursor for the insect antifeedant azadirachtin (Gagnon et al. 1994; Gagnon et al. 1995b). A series of monocyclic ketones were further explored and 2-alkylcyclopentanones and 3-substituted cyclobutanones were converted with often complementary enantioselectivity in comparison to transformations with whole cells of *Acinetobacter calcoaceticus*, which was finally attributed to 2-oxo-Δ3-4,5,5-trimethylcylopentenylacetic acid monooxygenase (Gagnon et al. 1995a; Grogan et al. 1993). These studies were performed with cells or cell-free extracts, which contained all three BVMOs or at least both diketocamphane monooxygenases. Even though separation of the distinct activities was tried by purification, the presence of impurities could not be excluded. Therefore, reproducible and reliable methods for separation and purification are required for the accurate characterization of these enzymes.

The availability of efficient cofactor recycling strategies for NADH-regeneration in BVMO-catalyzed oxidations, e.g. by the formate dehydrogenase from *Candida boidinii*, were also exploited. Moreover, coupling processes of horse liver alcohol dehydrogenase together with 2,5-DKCMO were used to produce optically active lactones starting from alcohol precursors (Gagnon et al. 1994; Gagnon et al. 1995b).

Several new BVMOs were investigated recently and while most of them refer to type I, which are FAD and NADPH-dependent, (Fraaije et al. 2005; Rehdorf et al. 2007; Völker et al. 2008; Rehdorf et al. 2009) only a few examples for FMN/NADH-containing type II BVMOs were investigated up to now. A reason might be the challenging overexpression of these enzymes in a heterologous host, since in contrast to type I BVMOs the oxygenating and dehydrogenase subunits are distinct proteins.

So far all characterization and biocatalytic experiments with 2,5-diketocamphane 1,2-monooxygenase were performed using large scale cultivations of the wild type strain *P. putida *NCIMB 10007 with subsequent multiple purification and separation steps of the involved enzymes. We report here the first recombinant overexpression of the oxygenating 2,5-diketocamphane 1,2-monooxygenase from *P. putida *NCIMB 10007 in *Escherichia coli *followed by simplified purification via affinity chromatography and characterization of the enzyme.

## Material and methods

### Enzymes, chemicals and media

Pfu^+^-polymerase was obtained from Roboclon (Berlin, Germany) and dNTPs from Roth (Karlsruhe, Germany). Restriction enzymes were obtained from New England Biolabs (Beverly, MA, USA). For SDS-PAGE analysis, the prestained PAGE ruler plus from Fermentas (St.Leon-Rot, Germany) was used. All other chemicals were purchased from Fluka (Buchs, Switzerland), Sigma-Aldrich (Munich, Germany) or Acros Organics (Geel, Belgium). For DNA-purification from PCR, the MinElute PCR-purification Kit by Qiagen (Hilden, Germany) was used. Furthermore the Miniprep Kit from Qiagen was used for plasmid purification. HisTrap 5 mL FF columns and Sephadex G25 were obtained by GE Healthcare (Uppsala, Sweden). The plasmid pET-28b(+) was from Novagen (Darmstadt, Germany). The BCA kit was purchased from Interchim (Montluçon, France).

### Amplification and cloning

**Figure 3:**
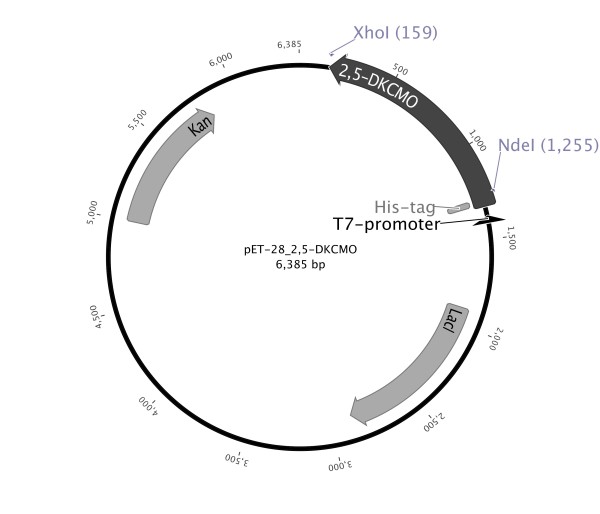
**Vector 2,5-DKCMO_pET-28 for expression of recombinant 2,5-DKCMO from *P. putida *NCIMB 10007 under control of T7 promoter in *E. coli *BL21**. The 2,5-DKCMO-gene was introduced using the sites of restriction endonucleases *Nde*I and *Xho*I for cloning.

Amplification of the 2,5-DKCMO gene was performed with chromosomal DNA containing the CAM-plasmid with oligonucleotides supplemented with restriction sites for *Nde*I at the N-terminus and *Xho*I at the C-terminus (*Nde*I_2,5-DKCMO_fw: 5'- GGAATTCATATGAAATGCGGATTTTTCCATACCCC-3'; 2,5-DKCMO_*Xho*I_rv: 5'- CCGCTCGAGTCAGCCCATTCGAACCTT-3'). After initial denaturation for 5 min at 95°C, the cycling program was followed for 25 cycles: 45 s, 95°C denaturation, 45 s, 58°C primer annealing, 70 s, 72°C elongation. The final elongation step was performed over 10 minutes at 72°C. The resulting 1092 kb fragment was digested with *Nde*I and *Xho*I and ligated into pET-28b digested with the same enzymes. The resulting plasmid with a N-terminal His-tag fusion was called pET-28_2,5-DKCMO (Figure 3).

### Bacterial strains and culture conditions

*P. putida *NCIMB 10007 (equivalent to ATCC 17453) was purchased from the German National Resource Center for Biological Material (DSMZ). For cultivation of *P. putida*, basal salt medium without antibiotics as described previously was used (Gagnon et al. 1994). *E. coli *cells were cultivated in terrific broth (TB) medium (12 g tryptone, 24 g yeast, 4 g glycerol in 1 L buffer autoclaved separately). Overnight cultures were grown in Luria Bertani (LB) medium (10 g tryptone, 5 yeast, 5 g NaCl in 1 L dest H_2_O). LB and TB media were supplemented with 100 μg/mL kanamycin.

Transformation of *E. coli *strain BL21-DE3 (Novagen, genotype: [95 F- *ompT hsdSB *(rB-mB-) *gal dcmrne131 *(DE3)]) with pET-28_2,5-DKCMO was carried out by the heat shock method described by Chung et al. (1989). Expression of recombinant 2,5-DKCMO in *E. coli *BL21 was performed by cultivation at 37°C to an OD_600 _of 0.5, than addition IPTG to a final concentration of 0.1 mM and shifting the culture to 20°C and 200 rpm for another 16 h of cultivation.

### Gene expression analysis

Gene expression analysis was performed with crude cell extract. Samples standardized to cell amount were taken during cultivation. Cells were harvested by centrifugation and resuspended in sodium phosphate buffer (50 mM, pH 7.5). Cell disruption was performed by FastPrep (40 s, 4 m/s; MP Biomedicals, Solon, OH, USA). For SDS-PAGE analysis, the supernatant was substituted with Laemmli buffer (Laemmli 1970). SDS-PAGE was carried out on 12% resolving gels. Proteins were stained with a Coomassie R250/G250 solution.

### Enzyme purification

Cells were harvested by centrifugation and resuspended in sodium phosphate buffer (50 mM, pH 7.5). Cell disruption was performed by a single passage through a French pressure cell. Recombinant 2,5-DKCMO was purified by affinity chromatography via N-terminal His-tag on an automated Äkta purifier system. After centrifugation of disrupted cells for 45 min at (10,000 × g), the supernatant with recombinant 2,5-DKCMO was added to the column. A 5 mL HisTrap FF crude column with bound Ni^2+ ^was equilibrated with sodium phosphate buffer (100 mM, pH 7.5) supplemented with 300 mM NaCl and 30 mM imidazole. After passing through of the crude extract, the column was washed with three column volumes of sodium phosphate buffer (100 mM, pH 7.5) supplemented with 300 mM NaCl and 30 mM imidazole followed by two column volumes of sodium phosphate buffer (100 mM, pH 7.5) supplemented with 300 mM NaCl and 60 mM imidazole to remove unspecific bound proteins. Elution was performed by adding three column volumes of 300 mM imidazole in sodium phosphate buffer (100 mM, pH 7.5) supplemented with 300 mM NaCl. Fractions of washing and elution steps were collected to analyze purity by SDS-PAGE. In order to remove imidazole and NaCl from the eluate, the pooled elution fractions were loaded to a 60 mL size exclusion column (Sephadex G25 matrix), which was equilibrated with sodium phosphate buffer (50 mM, pH 7.5) before. Proteins fractions were recognized via online absorption measurement at 280 nm and collected. Determination of protein content of purified and desalted protein as well as crude extract was carried out with the BCA-kit and a standard curve of BSA in the same buffer in a range of 2-0.005 mg/mL was used. Samples were measured in triplicates in three different dilutions.

### Biocatalytic reactions and GC analysis

For biocatalysis, His-tag purified 2,5-DKCMO, crude extracts of *E. coli *BL21 pET28_2,5-DKCMO cultivations and resting cells were used. Reactions were carried out in sodium phosphate buffer (50 mM, pH 7.5) Substrates were used in concentrations from 0.5-2 mM, the cofactor FMN was used at a final concentration of 0.3 mM. NADH was used in equimolar amounts to the substrate. Purified 2,5-DKCMO was employed in concentrations of 1.5-2 mg/mL, crude extracts in concentrations of 12-15 mg/mL. Incubation was performed in 24-well MTP at 800-1000 rpm. Sample volume was 1 mL. Extraction of substrates and products was performed by vortexing of samples with 600 μl and 400 μl of ethyl acetate subsequently. Samples were dried over anhydrous sodium sulfate. Separation of aqueous and organic phase was done by centrifugation. The organic solvent was evaporated in a vacuum centrifuge. 120 μL of fresh EtOAc was added, and samples were analyzed by GC-MS on a QP 2010 (Shimadzu Europa GmbH, Duisburg, Germany) with a BPX5 column (5% phenyl-/95% methylpolysilphenylene siloxane, SGE GmbH, Darmstadt, Germany). Injection temperature was set to 220 °C. Detection temperature for (+)-**1**, (-)-**1**, **13**, **14 **and **15 **was 60°C for 5 min followed by a gradient of 10°C/min to 180°C maintained for 3 min. Detection temperature for **16 **was 120°C. For **17**, 240°C for 5 min followed by a gradient of 2°C/min to 270°C was used and maintained for 5 min. **6**-**8 **were analyzed at 60°C. **9 **and **10 **were detected isothermal at 160°C. Detection temperature for **11 **was 90°C and for **12 **100°C.

Specific activity is given in units per milligram (U/mg) protein. One unit is defined as the amount of enzyme that catalyzes the oxidation of 1 μmol of substrate per minute.

## Results

### Cloning, expression and purification of 2,5-diketocamphane 1,2-monooxygenase

The 2,5-diketocamphane 1,2-monooxygenase (2,5-DKCMO) from *Pseudomonas putida *NCIMB 10007 is encoded on the CAM operon on the transmissible 230 kb CAM plasmid (Rheinwald et al. 1973). First chromosomal and plasmid DNA were isolated from the *P. putida *strain NCIMB 10007 cultivated with camphor as sole carbon source. The gene was then amplified by gradient PCR using gene specific primers derived from the corresponding gene [GenBank: AY450285]. The PCR product was afterwards ligated into the expression vector pET-28b fused to the N-terminal His-tag to allow a functional expression of the 2,5-DKCMO in *E. coli *and easy protein purification by affinity chromatography. The N-terminal tag was favored compared to the C-terminal tag, because our experience with 4-hydrocyacetophenonemonooxygenase (HAPMO) from *P. putida *JD1 indicated that BVMO-expression is decreased by the use of C-terminal tags (Rehdorf et al. 2009).

**Figure 4:**
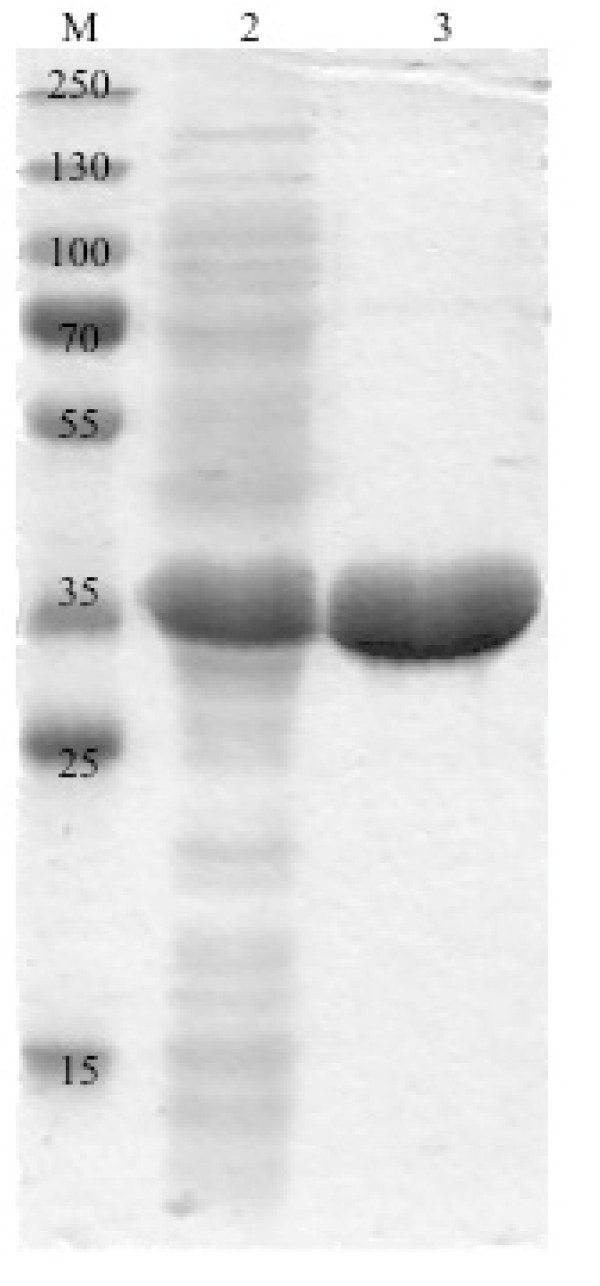
**SDS-PAGE analysis of 2,5-DKCMO: lane 1: marker: 150, 130, 100, 70, 55, 35, 25, 15 kDa; lane 2: crude extract (41 μg total protein); lane 3: purified protein (22 μg)**.

The utilization of *E. coli *BL21(DE3) as expression host yielded primarily soluble 2,5-DKCMO protein after 16 h cultivation at 20°C in TB medium, while cultivation at 30°C yielded in insoluble inclusion bodies (data not shown). SDS-PAGE analysis of crude cell extract led to a clear band at approx. 40 kD shown in Figure 4, which corresponds to the theoretical estimated molecular weight of 42.9 kD of the His-tagged protein.

After successful recombinant expression, a nickel-based affinity chromatography of the His-tagged protein and the subsequent removal of imidazole by size exclusion chromatography on a G25 column was performed and yielded pure protein (Figure 4, lane 3) with a purification factor of six (Table 1). The fractions containing purified protein were colorless, which confirmed previous studies, in which FMN is not covalently bound to the enzyme (Trudgill 1986).

**Table 1:** Purification of 2,5-DKCMO via nickel-based affinity chromatography and imidazole removal

Step	V [mL]	**Volumetric activity**^**a **^**[U/mL]**	**Activity**^**a **^**[U]**	**Protein amount**^**b **^**[mg/mL]**	Specific activity [mU/mg]	Yield [%]	Factor
Crude extract	30	0.0021	0.063	14	0.15	100	1

Purified and desalted	7	0.0015	0.0103	1.6	0.90	16	6

^a^Activity was determined towards (+)-camphor and analyzed by GC-MS.

Activity of the purified protein containing imidazole prior to the size exclusion chromatography could not be determined, since imidazole interferes with the used GC-MS column.

^b^Protein amount as determined by the BCA assay.

### Substrate specificity of 2,5-DKCMO

**Figure 5:**
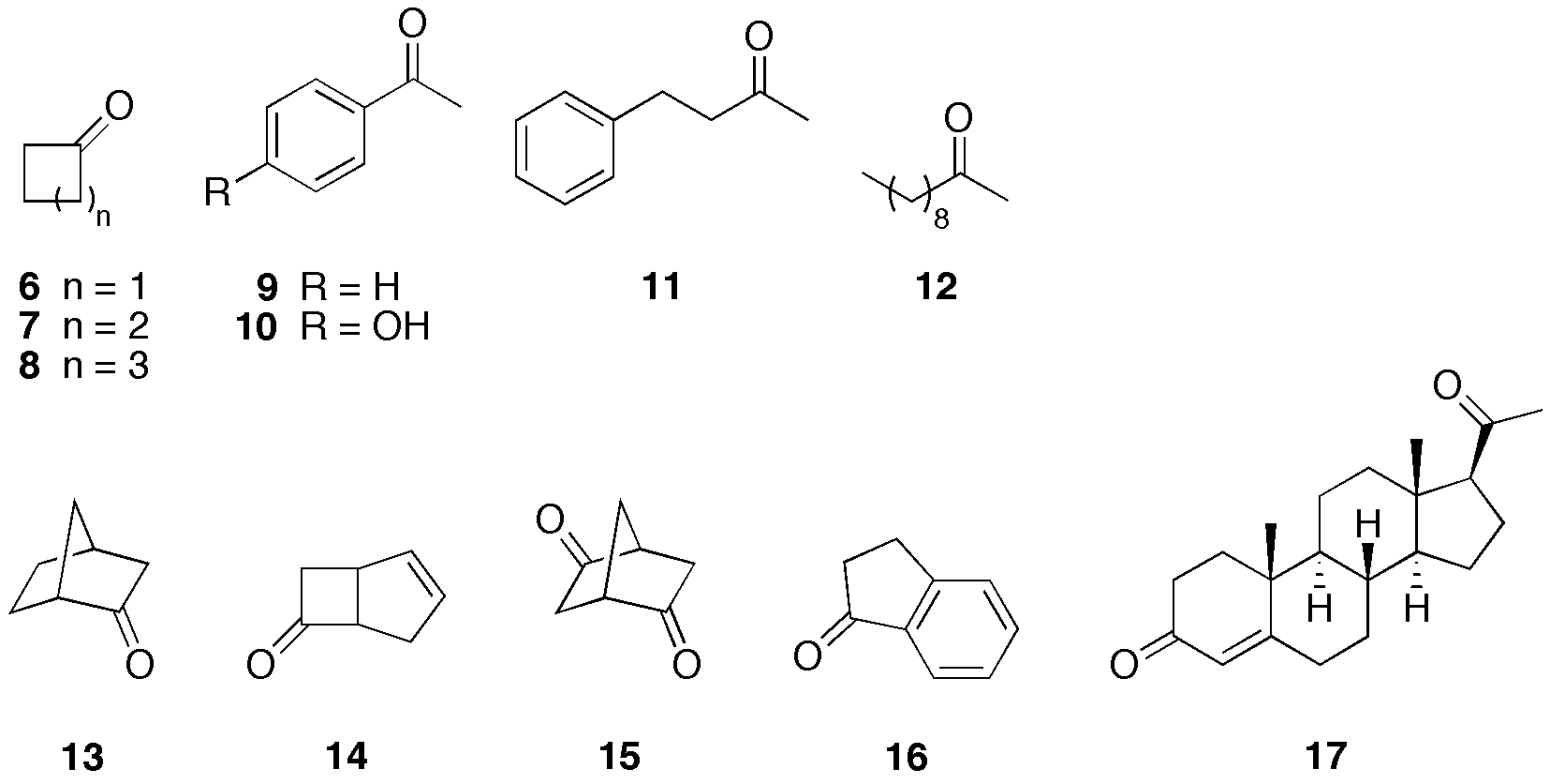
**Substrates used for 2,5-DKCMO-catalyzed Baeyer-Villiger oxidation**. 6-8 represent the monocyclic ketones, 9-11 substitute aromatic ketones, 12 served as an example for aliphatic and 13-16 for bicyclic ketones.

To determine the substrate specificity of 2,5-DKCMO a variety of compounds representing different classes of BVMO-substrates were investigated in biocatalysis experiments using the crude enzyme extract (Figure 5). Only bicyclic ketones were converted under the chosen conditions by the crude extract containing 2,5-DKCMO (Table 2). For all monocyclic ketones (**6**-**8**), aromatic ketones (**9**-**11**), the aliphatic 2-decanone (**12**) tested as well as for 1-indanone (**16**) and progesterone (**17**) no conversion could be determined. The biocatalysis with substrates, which were converted was further investigated using the pure enzyme and specific activities were determined in biocatalysis experiments in 1 mL scale with 2 mM of substrates at 25°C for 15 h (Table 2).

**Table 2:** conversion of several bicyclic ketones by crude cell extract and specific activities for pure 2,5-DKCMO

Substrate	**Conversion**^**a **^**[%]**	**Specific activity**^**b **^**[mU/mg]**
(+)-Camphor ((+)-**1**)	66	0.9

(-)-Camphor ((-)-**1**)	25	0.3

Norcamphor (**13**)	98	1.3

(±)-cis-bicyclo [3.2.0] hept-2-en-6-one (**14**)	100	1.4

(*R*,*R*)-bicyclo [2.2.1] heptane-2,5-dion (**15**)	94	0.06

^a^Conversion was determined by GC-MS analysis of biocatalysis samples with crude extract from *E. coli *cells overexpressing DKCMO at a substrate concentration of 1 mM after 23 h.

^b^Specific activity was calculated from conversion of substrate by pure enzyme determined by GC-MS at different points of time.

Interestingly, in our study (-)-**1 **was also converted by the 2,5-DKCMO, although purified enzyme isolated from wild-type strain cultivation was claimed to be specific for the (+)-enantiomer (Jones et al. 1993). As we have recombinantly produced the BVMO in the *E. coli *host, which does not have its own BVMO and the conversion of (-)-camphor was observed with crude cell extract as well as His-tag purified protein, we can only speculate whether the purified protein described by Jones et al. 1993 was indeed homogenous. Norcamphor (**13**) and (±)-cis-bicyclo [3.2.0] hept-2-en-6-one (**14**) were better accepted as substrates than camphor in general, and furthermore (*R*,*R*)-bicyclo [2.2.1] heptane-2,5-dion (**15**), which is structurally similar to the natural substrate 2,5-diketocamphane (**3**), is also converted. In addition, the conversion of **14 **was performed with resting cells expressing 2,5-DKCMO, where 11% conversion could be observed after 6 h of biocatalysis at 0.5 mM substrate concentration.

## Discussion

The oxygenating subunit of the 2,5-diketocamphane monooxygenase was successfully cloned and overexpressed recombinantly in *E. coli *as the heterologous expression host. Hence, this enzyme is now easy available at stable quality and protein engineering studies are possible for the first time. The purification using the N-terminal His-tag via nickel based affinity chromatography turned out to be efficient and fast. While in previous purifications of the enzyme from wild type cultivations, huge culture volumes were used, in this study drastically smaller amounts of heterologous culture is needed to produce comparable amounts of pure protein. Conrad et al. used a 10 L culture and obtained 240 mL crude extract to produce 52 mg of pure protein via a chromatography based purification protocol with three steps, which corresponds to a recovery of 15% (Conrad et al. 1965a). 28 years later Jones et al. were able to increase the purity and the yield up to 19.5%. From a 10 L culture volume 49 mg of pure enzyme were obtained (Jones et al. 1993). In this work 8 mg of pure protein were achieved out of a 400 mL culture, which highlights the advantages of recombinant expression and the fusion of an enzyme to a His-tag.

Previous studies on the purified protein determined a molecular size of 2,5-DKCMO of 78 kDa by native PAGE. Under denaturating conditions two identical subunits with a molecular weight of each 37 kDa were identified (Trudgill 1986). The estimated mass from the amino acid sequence of one subunit of 2,5-DKCMO is 40.7 kDa and fused to the His-tag 42.8 kDa. SDS-PAGE analysis of *E. coli *crude extract and pure protein resulted in protein bands corresponding to approx. 40 kDa, which corresponds to those molecular weights determined in earlier studies within a certain error range of the SDS-PAGE method.

Fractions containing 2,5-DKCMO collected by affinity chromatography turned out to be colorless. It was previously shown that FMN binding occurs non-covalently (Conrad et al. 1961), and therefore we assume that FMN is lost during the purification process. To achieve better stability of the enzyme, FMN was added to the protein solution immediately.

The requirement of non-heme Fe^2+ ^ions for oxygenating activity was intensively discussed in the past as well (Conrad et al. 1965a). Fe^2+ ^was thought to be essential for the generation of the active form of oxygen required for the BVMO reaction. In fact, there are no mechanistic requirements for transition metal-ions in the enzyme, which could also be confirmed by the availability of BVMO-activity of pure protein in the absence of Fe^2+ ^within this study.

In this work, recombinant expression and purification of 2,5-DKCMO, an oxygenating subunit, led to a "dehydrogenase-missing" pure protein and it could be shown that the enzyme is still able to oxidize bicyclic ketones. Previously, marginal BVMO-activity was obtained although no NADH dehydrogenase was detectable in the final preparation of 2,5-DKCMO, which was finally reasoned with impurities or the fact that the oxygen component is able to operate as its own NADH dehydrogenase in presence of FMN and remove electrons from NADH to catalyze the reaction (Trudgill 1986). Low activities of purified oxygenating component were observed earlier as well and were explained by a weak coupling of the mentioned subunits *in vitro *(Conrad et al. 1965b).

We also observed that oxygenating activity of 2,5-DKCMO expressed in *E. coli *is higher in the crude extract or whole cell approaches when compared to pure protein. This fact might be explainable by several components of *E. coli *cells that may substitute the missing NADH dehydrogenase. Coexpression experiments with a suitable NADH dehydrogenase may further improve the activity of 2,5-diketocamphane 1,2-monooxygenase considerably and could thus generate valuable catalysts for organic synthesis providing access to industrial valuable precursors for e.g. azadirachtin.

Regarding the requirement for cofactor regeneration in larger scale applications, the 2,5-DKCMO might also be used in whole cell approaches with the expression system introduced in this report.

## Abbreviations

FMN: flavin mononucleotide; NADH: nicotinamide adenine dinucleotide; BVMO: Baeyer-Villiger monooxygenase; 2,5-DKCMO: 2,5-diketocamphane 1,2-monooxygenase

## Competing interests

The authors declare that they have no competing interests.
